# In situ and bio-green synthesis of silver nanoparticles immobilized on zeolite as a recyclable catalyst for the degradation of OPDs

**DOI:** 10.1038/s41598-024-51271-9

**Published:** 2024-01-11

**Authors:** Fujiang Zhou, Danfeng He, Guojian Ren, Hossein Yarahmadi

**Affiliations:** 1 College of Science, Qiongtai Normal University, Haikou, 571100 Hainan China; 2 Key Laboratory of Advanced Materials of Tropical Island Resources, Ministry of Education, School of Chemistry and Chemical Engineering, Hainan University, Haikou, 570228 Hainan China; 3 Department of Chemical Engineering, Sirjan University of Technology, Sirjan, Iran

**Keywords:** Biogeochemistry, Environmental sciences, Heterogeneous catalysis

## Abstract

In this study, silver nanoparticles (Ag-NPs) were synthesized using a green and biologically inspired approach by utilizing reducing compounds from *Thyme plant leaves*. Zeolite was used to immobilize the synthesized Ag-NPs (Ag@Z). The modified Zeolite served as a catalyst for the reduction reaction of various organic pollutant dyes (OPDs) including 4-nitrophenol (4-NP), 4-nitroaniline (4-NA), methylene blue (MB), and methyl orange (MO) with sodium borohydride. The degradation of OPDs was monitored by measuring changes in their maximum absorption wavelength intensity. A thorough examination of multiple parameters (catalyst, silver and sodium borohydride dosage, yield degradation, and reaction time) was carried out to identify the optimized conditions for the degradation of OPDs. The results showed that the Ag@Z catalyst achieved an efficiency of over 93% in less than 10 min for the degradation of OPDs. The recoverability and reusability of the catalyst were examined, revealing a partial loss in efficiency after four recovery stages. Structural analysis using XRD, SEM, and TEM techniques confirmed the characteristics and morphology of the synthesized catalyst.

## Introduction

The remarkable catalytic activity of small metallic nanoparticles (NPs), including Au, Rh and Ag, has made them highly desirable for the efficient treatment of toxic pollutants^1–3^. In comparison to their bulk materials, ultrafine metallic NPs offer distinct advantages, such as a large specific surface area (SSA) and a significant amount of active atoms^4^. Nevertheless, the high surface area of noble metal NPs makes them prone to aggregation, leading to a significant decline in their catalytic performance^2,4^.

To surpass this challenge, one of the most efficient strategies is to stabilize the ultrafine metallic NPs within diverse carrier materials, particularly porous materials, to prevent their aggregation^5,6^. Previous studies have shown that confining metallic NPs within porous silica, metal oxides, carbon nanotubes (CNTs), metal and covalent organic frameworks (MOFs and COFs), can greatly enhance their stability^7–9^. It is noteworthy that the immobilization of nanoparticles on these substrates not only facilitates their recovery but also enhances the overall process efficiency. This, in turn, leads to cost and time reductions, which are crucial considerations from both economic and environmental perspectives^5–7^.

However, it is important to note that while carbon and metal oxides, as well as MOFs and COFs, have been extensively studied as porous materials, they have certain limitations. Metal oxides and CNTs generally exhibit low SSAs, while MOFs and COFs are often unstable in acidic (or alkaline) conditions, and the synthesis of COFs typically involves hazardous solvents, reagents, materials, and energy-intensive reaction conditions^10–13^. Therefore, there is a need to explore and develop suitable alternative supports. In comparison, zeolite materials offer distinct advantages as supports for ultrafine noble metal NPs. They possess a large SSA for efficient NP loading, a rich pore structure to inhibit NP accumulation, and a low-cost preparation process^14–16^.

Zeolite, a crystalline alumino-silicate material, has garnered significant attention as a promising support in catalytic applications^17^. This is mainly due to its unique structural characteristics, including well-defined pores, cavities, and channels, as well as its remarkable thermal and chemical stability^17,18^. As a carrier or encapsulating shell, zeolite is commonly utilized for the synthesis of metal nano-catalysts^17^. This strategy ensures the protection of catalytically active species while also exerting constraints that can influence the specificity of the catalytic reaction^17,19^.

The porous surface channels of the zeolite sites can serve as a deposition site for the active metal NPs assembly. However, it is important to consider that active sites may also develop within the pores of the shell, thereby imparting similar characteristics to conventional catalyst support materials. In addition, utilizing mesoporous zeolites as a platform for immobilizing metallic NP catalysts causes a decline in the number of active contact sites and the catalytic efficiency of the metallic NPs. This decrease can be attributed to the confinement of these metallic NPs within multiple zeolite structures, which restricts their interaction with the external environment^20,21^.

Noble metallic NPs are prepared using various techniques, including physical, chemical, and biological methods, for controlling properties such as the morphology, size and stability of synthesized structures. For example, several methods have been reported for the synthesis of silver nanoparticles, such as radiation-induced chemical reactions, reduction reactions, thermal decomposition, chemical vapor deposition, sonochemical route, co-precipitation, combustion, chemical spray pyrolysis, etc.^22^. However, these synthetic methods are widely associated with several drawbacks due to their long reaction times, complex synthesis steps at high temperatures, expensive equipment, and high synthesis costs^23^. Moreover, most of the mentioned methods are not environmentally friendly^24^. Therefore, the approach of “green chemistry for metallic NPs” is considered the best method^25,26^.

Green chemistry is proposed as an alternative approach to eliminate or reduce the drawbacks associated with these methods. Green chemistry is an innovative solution for minimizing the use of hazardous and toxic chemicals in the synthesis of metal nanoparticles^27^. It involves the use of environmentally friendly materials that offer compatibility and eco-friendliness advantages. Green synthesis, using plant and microorganism extracts as reducing and stabilizing agents, has gained significant attention recently due to its utilization of mild experimental conditions, including temperature, pH, and pressure.

Plant extracts are convenient to use, non-toxic, and can be processed with simple protocols. The bioactive compounds found in plant extracts, such as saponin, tannins, flavonoids, phenolic acids and etc., act as effective reducing agents for nanoparticle synthesis. These compounds have the ability to donate hydrogen and quench singlet oxygen. Plant-assisted green synthesis is a more suitable alternative to physico-chemical methods due to the redox activities of these bioactive compounds^28^. Natural extracts such as plants, fungi, yeasts, algae, and microorganisms are used in the process of biological synthesis. So far, different and diverse plant extracts, such as aloe-vera, pepper, mint, green tea, walnut shell, eucalyptus, lemon, and thyme, have been reported and utilized for the production of noble metal NPs through biosynthesis^26–29^. The presence of reducing compounds in plant extracts leads to the reduction of metal ions into NPs^30^. Therefore, the biosynthesis method is recognized as a cost-effective, sustainable, green and low-risk approach for the production of well-known noble metallic NPs.

In recent years, metal nanoparticles immobilized on zeolites have gained considerable attention due to their diverse applications. Notably, Wattanakit et al. successfully employed gold-supported nanoparticles on zeolites to produce furan dicarboxylic acid^31^. The use of copper NPs immobilized on zeolites was explored as an efficient and recyclable catalyst for the degradation of OPDs^32^. Supported Ag-NPs on zeolites was utilized to detection of H_2_O_2_, glucose^33^, carbon monoxide^34^ and synthesis bis-cyclohexenones^35^. Also, Ag-NPs have been extensively studied and utilized in various research areas, including pharmaceuticals and antibacterial agents^36,37^. One of the key advantages of immobilized Ag-NPs is their ability to catalyze the degradation of OPDs^38–41^. This property makes them highly desirable for the development of efficient and sustainable materials for wastewater treatment and environmental remediation.

Combined with the above analysis, in this study, we adopted a green and biological approach to synthesize Ag-NPs, utilizing *Thyme* extract as the reducing agent. These nanoparticles were subsequently immobilized on zeolite for further investigation. The catalytic efficiency of the immobilized Ag-NPs was thoroughly examined, and its recyclability and reusability were assessed to determine its potential for sustainable applications.

## Instruments, materials and methods

### Chemical reagents and characterization methods

We obtained all the chemicals (sodium hydroxide, alumina trihydrate, sodium silicate, silver nitrate, methyl orange (MO), methylene blue (MB), 4-Nitrophenol (4-NP), and 4-Nitroaniline (4-NA)) from Merck and Sigma-aldrich chemical companies. These chemicals were of analytical grade and used without any further purification. Thyme, commonly used domestically and medicinally in Iran, was collected for this research project under the supervision of the Chemical Engineering Department at Sirjan University of Technology. The collection took place in May 2023, during the spring season, and followed the licenses issued by the Natural Resources and Watershed Management Organization of Iran. Stringent adherence to institutional, national, and international guidelines and legislation was ensured throughout the process. Thyme plant leaves were collected and identified by M. Yarahmadi from a mountainous region between Sirjan and Hajiabad, specifically in Chah-Konar village located 60 km south of Sirjan in southern Iran. A voucher specimen (Herbarium no: 35314, FUMH) was preserved in the Herbarium of the School of Agriculture, Ferdowsi University. In this study, we investigated the crystal structure (X-Ray diffraction analysis (XRD)) of prepared A-zeolite and Ag@Z using the D8-ADVANCE XRD instrument (Bruker, Germany) and Cu-Kα radiation. The scanning range was set from 10 to 80 degrees at a rate of 2 degrees per min. The surface morphology and Energy-dispersive X-ray spectroscopy analysis (EDS) of the synthesized samples were recorded by a TESCAN BRNO Field-Emission Scanning Electron Microscopy (FE-SEM) at a voltage of 15.0 kV. Transmission electron microscopy (TEM) images obtained from the Philips EM 208S instrument were used to further examine the sample morphology. Additionally, the progress of OPD reduction reactions was monitored using a UV–vis spectrophotometer equipped with a quartz cell, and absorption measurements were recorded to evaluate changes over time. FT-IR was used to characterize the Ag-NPs and their spectra were recorded in the range of 400–4000 (KBr, cm^–1^) wavenumbers on a JASCO 6300 spectrophotometer.

### Preparation of thyme leaves extract

The collected *Thyme plant leaves* were thoroughly washed with deionized water and subsequently dried at 25 °C using an incubator. After drying, the leaves were finely powdered using a mortar. A 10.0 g portion of the powder was then boiled in 250 mL of water for a duration of 30 min, resulting in a noticeable change in the solution's color to a light yellow hue. The extract was carefully filtered through Whatman No. 1 paper and stored at room temperature for subsequent analysis.

### Preparation of zeolite and Ag@Z

A validated method was employed to prepare the Zeolite utilized in this study^42^. With minor modifications and the implementation of an in-situ synthesis approach, the synthesized Ag-NPs were immobilized with various ratio on zeolite achieved using established methodologies^43,44^. In order to synthesize Ag@Z, a 250 ml round bottom flask fitted with a water condenser was utilized. The procedure involved the addition of 10–20 ml of *Thyme leaves extract* and 1.0 g of as-synthesized zeolite to aqueous AgNO_3_ solution (20–150 ml, 0.02 M) and the resulting mixture were stirred in darkness conditions at 100 °C for 8 h. After the reaction was finished, the solid product was filtered and subjected to overnight heating at 80 °C under oven conditions. These prepared samples (Table 1) were subsequently employed to examine its catalytic activity.

**Table 1:** Preparation of Ag@Z using various ratios of silver nanoparticles.

Entry	*Thyme* (ml)	AgNO_3_ (ml, 0.02 M)	Zeolite (gr)	Catalyst (Ag@Z)
1	10	20	1	Ag@Z-1
2	10	40	1	Ag@Z-2
3	10	60	1	Ag@Z-3
4	10	80	1	Ag@Z-4
5	10	100	1	Ag@Z-5
6	20	120	1	Ag@Z-6
7	20	150	1	Ag@Z-7

### Catalytic degradation of OPDs

In order to evaluate the efficiency of the synthesized catalysts (Table 1, entries 1–7), which consisted of Ag-NPs coated on Zeolite (Ag@Z), a catalytic degradation test was conducted. This test aimed to assess the catalyst's capability in model OPDs, namely MB, MO, 4-NP, and 4-NA. The experimental procedure involved the addition of 5–10 mg of Ag@Z to a 100 mL aqueous solution containing 50 mg/L of various OPDs. To establish effective interaction between the catalyst and OPDs, the mixture was stirred for 0.5 h. This stirring process aimed to ensure proper adsorption of OPDs onto the catalyst surface and achieve adsorption–desorption equilibrium. Subsequently, a NaBH_4_ solution with a concentration of 0.1 M (10–50 mL) was introduced into the reaction mixture. The reaction mixture was continuously stirred at room temperature using a magnetic stirrer throughout the entire duration of the experiment. Regularly, 2.5 mL samples of the reaction solution were extracted and promptly diluted with 5 mL of water. Following the reaction, the mixture was subjected to centrifugation to separate the catalyst from the solution. The extent of color degradation was assessed using UV–vis spectroscopy, with absorbance changes at the respective maximum wavelength being measured (Table 2). The primary aim was to optimize parameters of catalytic OPDs degradation, including reaction time, sodium borohydride dosage, catalyst efficiency and its recovery and reusability. This investigation sought to enhance the overall effectiveness of the catalyst in degrading the OPDs.

**Table 2:** The maximum wavelength of the dyes used in catalytic degradation reactions in the presence of NaBH_4_ and Ag@Z catalyst.

OPDs	MB	4-NP	4-NA
λ_max_ (nm)	663	400 (317)	380

## Result and discussion

### The role of Thyme leaves extract for the reduction of Ag ions

**Figure 1:**
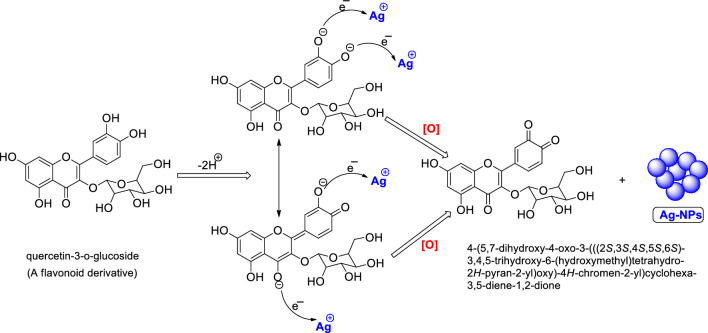
Depiction of the proposed mechanism for the synthesis of Ag-NPs through the reduction of silver ions by quercetin-3-o-glucoside as the model phytochemical active reducing agent.

Different parts of plant extracts, have been found to be abundant in phytochemicals, particularly phenolic compounds and flavonoids^45,46^. The thyme extract utilized in this study contains a significant quantity of metabolites that consist of aromatic rings with reactive hydroxyl (-OH) groups, which are believed to act as both reducing and capping agents^47^. The proposed mechanism for synthesizing Ag-NPs using the thyme extract is summarized in Fig. 1. In brief, AgNO_3_ molecules in the aqueous environment dissociate into silver ions (Ag^+^) and nitrate ions (NO^3–^). The release of protons from flavonoid molecules facilitates the reduction of two silver ions, causing them to cluster together and form the Ag-NPs^46^.

### Efficiency of thyme leaves extract to reduction of silver ions

The effectiveness of the *Thyme leaves extract* solution in reduction reaction of silver ions was investigated by AgNO_3_ solution (0.02 M). A 250 ml round bottom flask fitted with a water condenser was utilized. Specifically, 10–20 ml of the extracted solution was added to AgNO_3_ solution (20–150 ml, 0.02 M) and the resulting mixture was stirred magnetically (1200 rpm) in darkness conditions at 100 °C for 8 h. The concentration of silver ions in the solution was analyzed using the maximum wavelength of silver nitrate through UV–vis analysis^48^. As the concentration of silver ions decreased, the peak intensity at 300 nm was decreased, while the peak absorption intensity associated with Ag-NPs was simultaneously increased. Under the specified conditions, it was found that 10 ml of the *Thyme leaves extracted* solution had the capacity to reduce 0.0012 mol of silver ions.

### The most effective Ag@Z for degradation of OPDs

In the initial study, the objective was to determine the most effective Ag@Z catalyst for the degradation of OPDs. Several Ag@Z catalysts with varying silver ratios (Table 3, entries 1–7) were examined for their effectiveness in the degradation of 4-nitrophenol. A solution of 4-nitrophenol (50 ml, 50 ppm) was subjected to degradation reactions along with NaBH_4_ solution (50 ml, 0.1 M) in the presence of 10 mg of Ag@Z. The results were assessed after a reaction time of 120 min in order to evaluate the outcome of the experiment that presented in Table 3. Based on the obtained data, it is recommended to employ Ag@Z catalyst with higher Ag-NPs ratio to enhance the efficiency of the 4-NP degradation reaction and Ag@Z-5 was selected as optimized catalyst for degradation reaction of OPDs (Table 3, entry 5). Further characterization and investigations on the kinetics of the degradation reaction were suggested using Ag@Z-5 Catalyst.

**Table 3:** Degradation of 4-NP in the presence of various Ag@Z catalyst*.

Entry	Catalyst	Degradation (%)
1	Ag@Z-1	56
2	Ag@Z-2	74
3	Ag@Z-3	81
4	Ag@Z-4	90
5	Ag@Z-5	99
6	Ag@Z-6	99
7	Ag@Z-7	99

*Conditions: 4-NP (50 ml, 50 ppm), NaBH_4_ (50 ml, 0.1 M), catalyst (10 mg), 120 min. at room temperature.

### Mineralogical data and crystalline structure of Ag@Z-5

**Figure 2:**
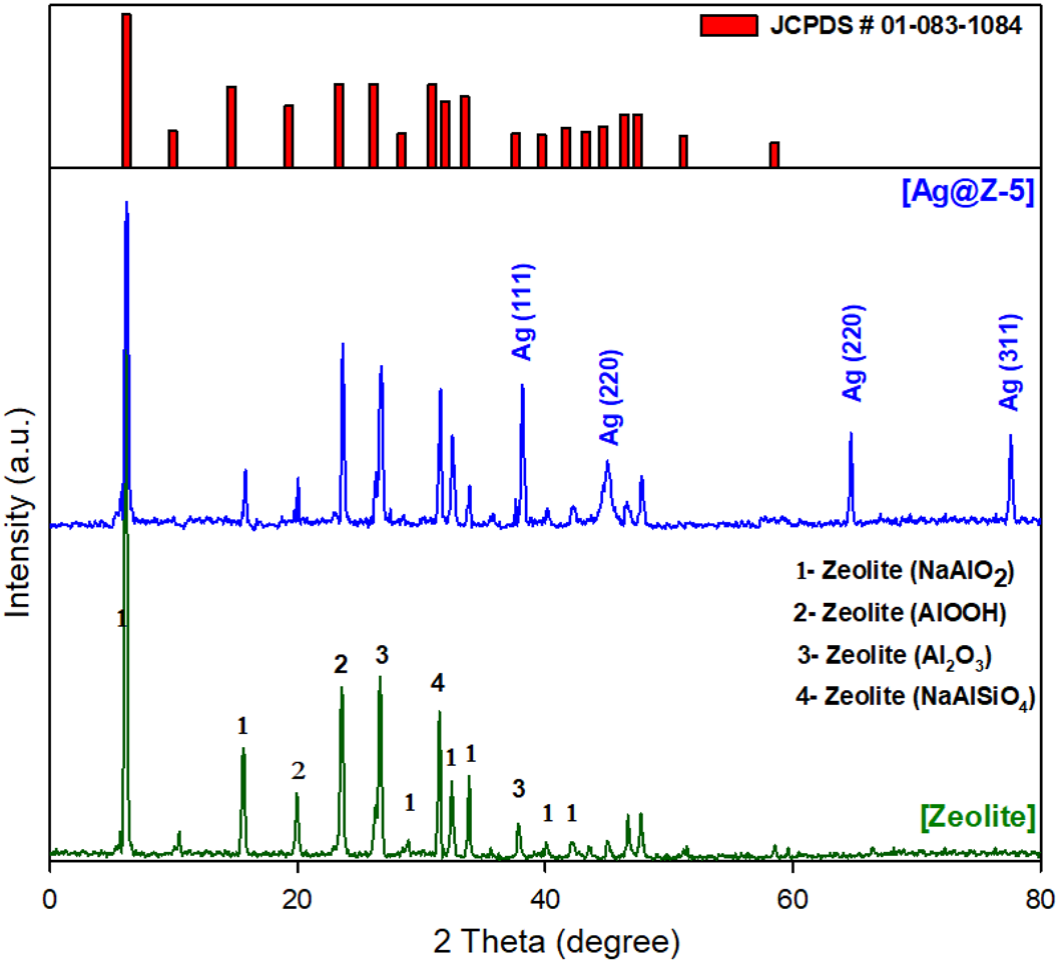
XRD pattern of zeolite and Ag@Z-5.

The crystallographic analyses presented in Fig. 2 encompass both the synthesized mesoporous materials (zeolite and Ag@Z-5). The XRD peaks are evident at 2θ = 6.1°, 15.5°, 19.9°, 23.5°, 26.8°, 31.4° and 37.8° for the zeolite exhibit a compelling concurrence with the literature, signifying the successful formation and synthesis of zeolite^42^. Subsequently, an evaluation of the XRD scattering was conducted following the deposition of Ag-NPs onto the surface of the initial zeolite, in comparison to the scattering pattern of the pristine zeolite. This comparative examination revealed a significant similarity between the two XRD patterns, excluding the emergence of novel peaks at points 2θ = 38.1°, 44.8°, 64.5° and 77.5°, which can be ascribed to the presence of the Ag-NPs. It is worth noting that the presence of silver metal is not observed in the initial zeolite phases (zeolite) after the immobilization of nanoparticles on zeolite. Instead, the silver metal is only visible in new phases (111, 220 and 311). This indicates that the incorporation of silver nanoparticles into the zeolite matrix does not affect the crystal structure of the zeolite. However, a comparison of the two XRD patterns reveals a decrease in peak intensities upon the incorporation of silver nanoparticles into the zeolite structure. This decrease can be attributed to a reduction in zeolite crystallite size and non-uniformity in crystal size resulting from the incorporation process^49,50^. Furthermore, the XRD analysis of Ag@Z-5 reveals that the peaks closely match those of the initially synthesized zeolite. It is noteworthy that the immobilization of Ag nanoparticles does not result in any noticeable shift in the peak positions. This suggests that the incorporation of Ag-NPs has minimal impact on the crystalline structure of the zeolite, affirming the structural stability of zeolite.

The calculation of the crystalline size is based on the half-height width of the diffraction peak observed in the XRD pattern. By plotting the XRD data, the particle size of the Ag@Z-5 can be determined using Scherrer’s equation:

$${D}_{p}=\frac{k\times \lambda }{{\beta }_{1/2}\times cos \theta }.$$

**Figure 3:**
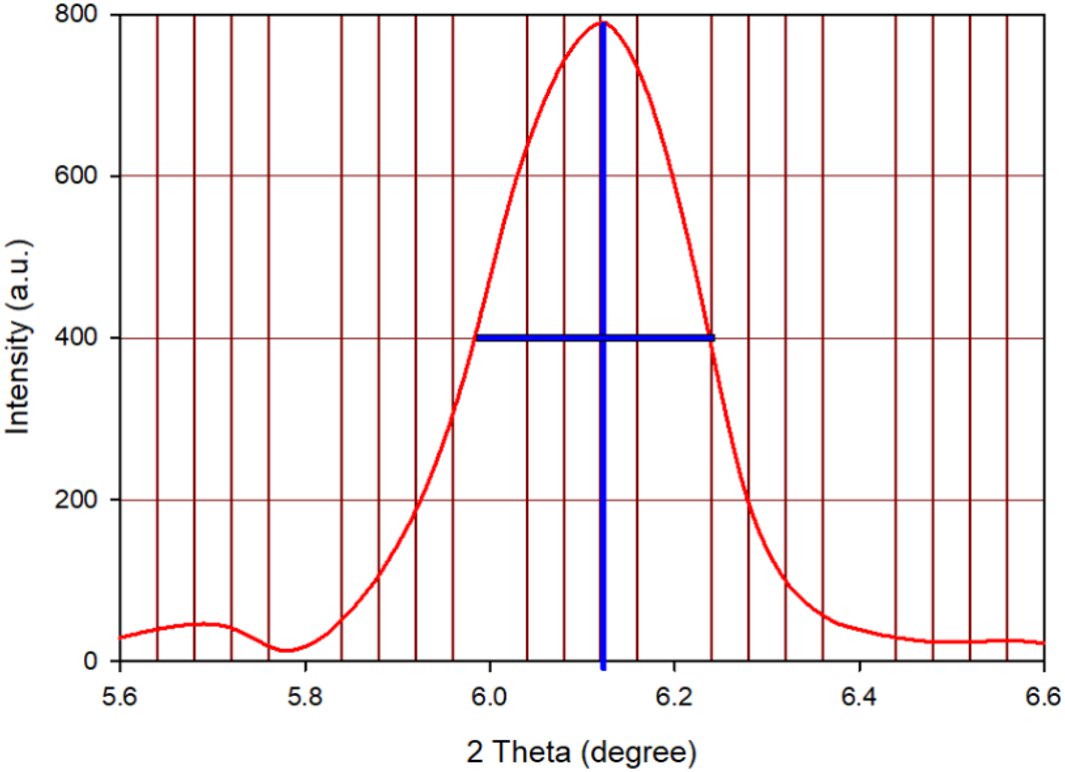
XRD image of Ag@Z-5 particles showing magnified peak.

Scherrer’s equation is employed to calculate the particle size in this context where *k* is the Scherrer constant with value from 0.9 to 1 (shape factor), λ is X-ray wavelength (0.154 A^o^), β_1/2_ is the line broadening at half the maximum intensity and *θ* is the Bragg angle (Fig. 3).

The particle size of Ag@Z-5 was determined to be 29.48 nm according to the Scherrer’s equation:$k=0.9, \lambda =0.154 {A}^{o},{\beta }_{1/2}={(6.24-5.97)}^{o}={0.27}^{o}=0.00471 rad.;$

$${D}_{p}=\frac{k \times \lambda }{{\beta }_{1/2} \times Cos \theta } = \frac{0.95\times 0.154 {A}^{o}}{0.00471\times cos \left(\frac{6.12}{2}\right)} = 31.11 \,\,nm.$$

### Morphology and elemental composition of Ag@Z-5

**Figure 4:**
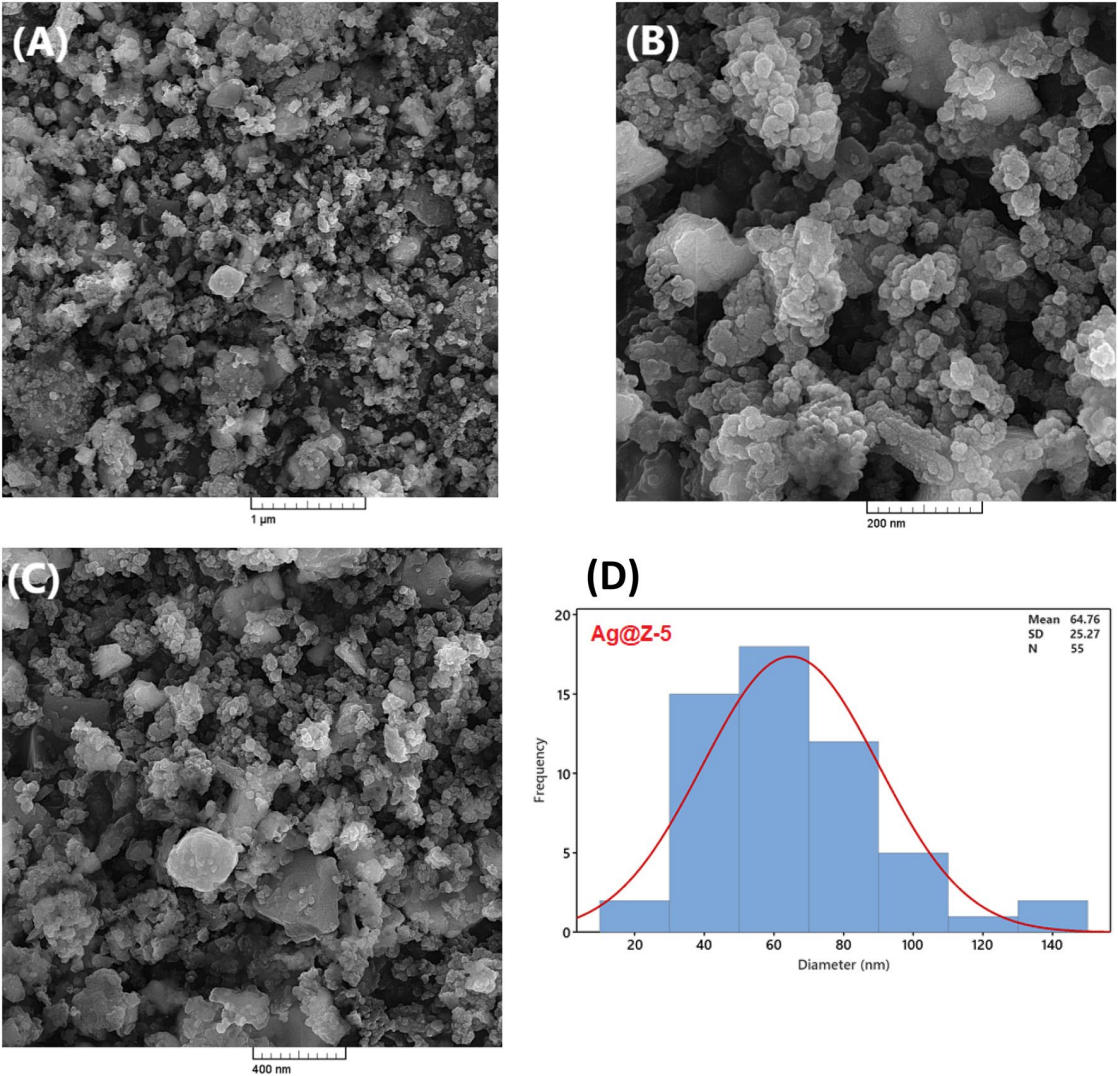
FE-SEM images (**A**–**C**) and corresponding histogram of particle size distribution (**D**) for Ag@Z-5.

The size and morphology of the Ag-NPs in the Ag@Z-5 were examined using FESEM and TEM techniques. The FESEM images shown in Fig. 4A–C illustrate the morphology of the Ag@Z-5. It can be observed that the Ag nanoparticles have a spherical shape and a narrow diameter distribution. In this investigation, the morphology of the synthesized Ag@Z-5 composite samples were examined using SEM and TEM techniques. The SEM image (Fig. 4) vividly illustrates the nano-particulate nature of Ag@Z-5, presenting discernible inclined aggregations and variations in particle sizes. Furthermore, the image offers a comprehensive visualization of the geometric and octahedral structure of Ag@Z-5. The SEM images not only showcase the nano-particulate nature of Ag@Z-5 and its distinct aggregations but also provide insights into its geometric and octahedral structure. Additionally, the analysis of particle size distribution reveals a mean diameter of 64.76 ± 0.5 nm for Ag@Z-5, furnishing quantitative data on the distribution of particle sizes (Fig. 4D).

**Figure 5:**
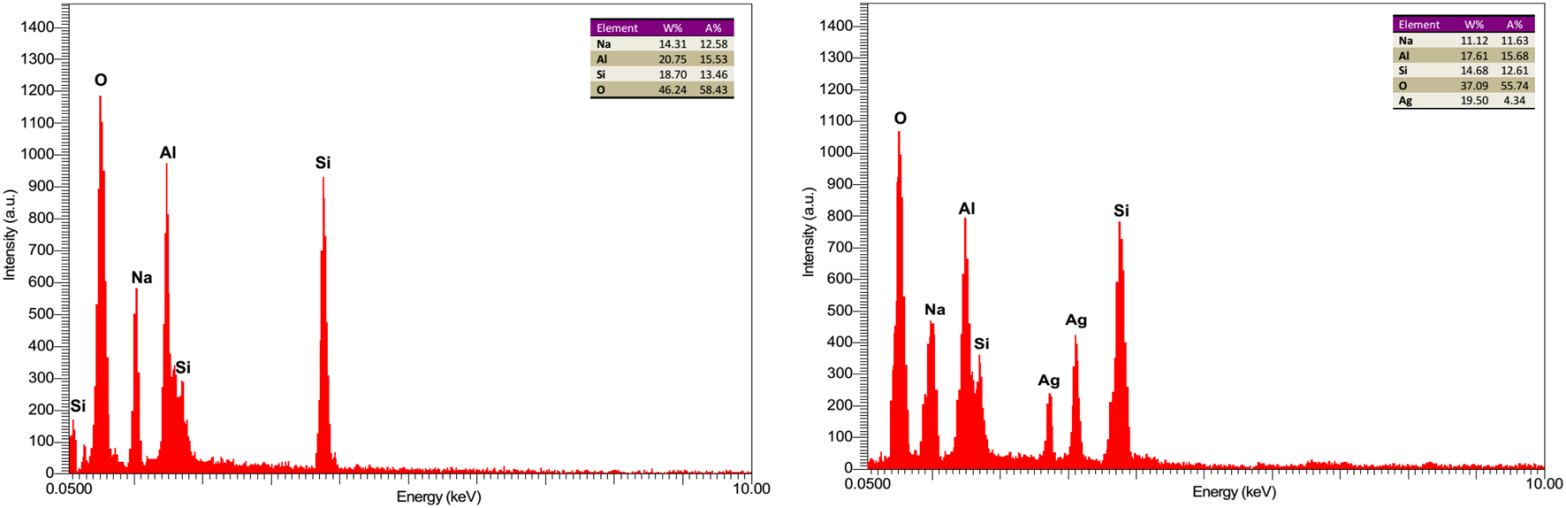
EDS analysis of synthesized zeolite (left) and Ag@Z-5 (right).

The chemical composition of the synthesized Zeolite and Ag@Z-5 were examined using energy dispersive spectroscopy (EDS) and the results confirmed the presence of Na, Al, Si, O and silver (Fig. 5).

The table inserted in the EDS pattern displays the quantitative analysis of the constituent elements in Zeolite and Ag@Z-5. The EDS analysis also revealed that the Zeolite consisted of 14.31% weight of Na, 20.75% weight of Al, 18.70% weight of Si, and 46.24% weight of O (Fig. 5, left). Also, Ag@Z-5 consisted of 11.12% weight of Na, 17.61% weight of Al, 14.68% weight of Si, 37.09% weight of O and 19.50% weight of Ag in EDS analysis (Fig. 5, right). Furthermore, the atomic percentages of elements were determined as follows in Fig. 5.

**Figure 6:**
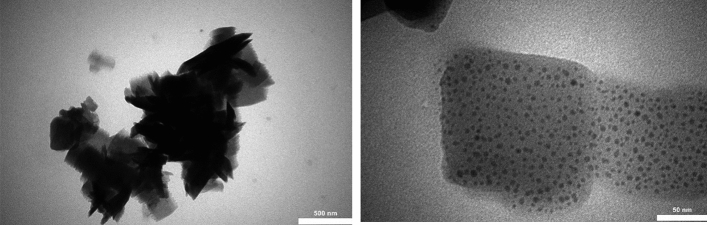
TEM images of synthesized Ag@Z-5.

Figure 6 presents the TEM analysis of Ag@Z-5, providing valuable insights into its structural properties. The TEM image exhibits a uniform distribution of nano-metallic units within the zeolite, emphasizing their homogeneous nature. Moreover, the direct interaction between Ag-NPs and zeolite leads to the formation of Ag nanoparticles on the surface of zeolite. Remarkably, the decorated surface of zeolite showcases the presence of multiple Ag nanoparticles. This contact interaction between Ag-NPs and zeolite holds significant potential for facilitating efficient electron transfer between the electronic surfaces, resulting in enhanced catalytic reaction efficiency.

### Optimal conditions for the degradation of OPDs

The utilization of catalysts plays a vital role in enhancing the speed and efficiency of chemical processes. However, it is important to note that simply increasing the dosage of catalysts does not always result in improved efficiency and can lead to unnecessary economic wastage. Therefore, it becomes imperative to optimize the dosage of catalysts to ensure both environmental protection and cost-effectiveness. In this study, we initially focused on investigating the significance of key compounds in the degradation of organic dyes, aiming to determine the optimal reaction conditions (as presented in Table 4). Each dye type (MB, 4-NP, 4-NA, and MO) was individually evaluated to establish the most suitable conditions. To evaluate the degradation process, 50 mL of dye solution with a concentration of 50 ppm was subjected to measurement and assessment in all reactions. The initial phase involved examining the degradation of the dye in the presence of Ag@Z-5 and NaBH_4_ separately, as well as their simultaneous presence.

**Table 4:** The key role and simultaneous presence of Ag@Z-5 and NaBH_4_ in the catalytic degradation of OPDs^a^.

Entry	Catalyst (mg)	NaBH_4_ (0.1 M, mL)	MB Deg. (%)^b^	MO Deg. (%)	4-NP Deg. (%)	4-NA Deg. (%)
1	Zeolite (5.0)	–	–	–	–	–
2	Zeolite (5.0)	50	7	–	5	–
3	Ag@Z-5 (5.0)	–	–	–	–	–
4	Ag@Z-5 (5.0)	50	98	97	99	94
5	–	50	6	–	5	–
6	AgNO_3_ (5.0)	50	99	96	98	95
7	AgNO_3_ (5.0)	–	–	–	–	–
8	zeolite(5.0) + AgNO_3_ (5.0)	–	–	–	–	–

^a^Conditions: All reaction efficiencies were determined by measuring the degradation of OPD solution (50 mL, 50 ppm) after 120 min of reaction time.

^b^Deg. degradation.

The results obtained from the experiments clearly indicate a notable decrease in the degradation of dyes when either NaBH_4_ or Ag@Z-5 was absent, especially as the reaction time extended. These findings underscore the indispensability of both NaBH_4_ and Ag@Z-5 in achieving satisfactory efficiency in the degradation process of organic dyes. Moreover, it can be inferred that the presence of silver metal plays a pivotal catalytic role in the reaction of dye degradation. In the absence of a catalyst, the reaction fails to occur, potentially due to a significant disparity between the energy levels of the reducing agent (NaBH_4_) and the acceptor (dye compound).

The energy levels of these species remain separated, and the absorption intensity at λ_max_ remains relatively unchanged, indicating limited reduction and degradation of color. However, the addition of the Ag@Z-5 composite as a catalyst in the reaction mixture generates an intermediate electronic level due to the presence of silver metal. This facilitates the transfer of electrons between energy levels by reducing the required distance for electron transfer.

**Figure 7:**
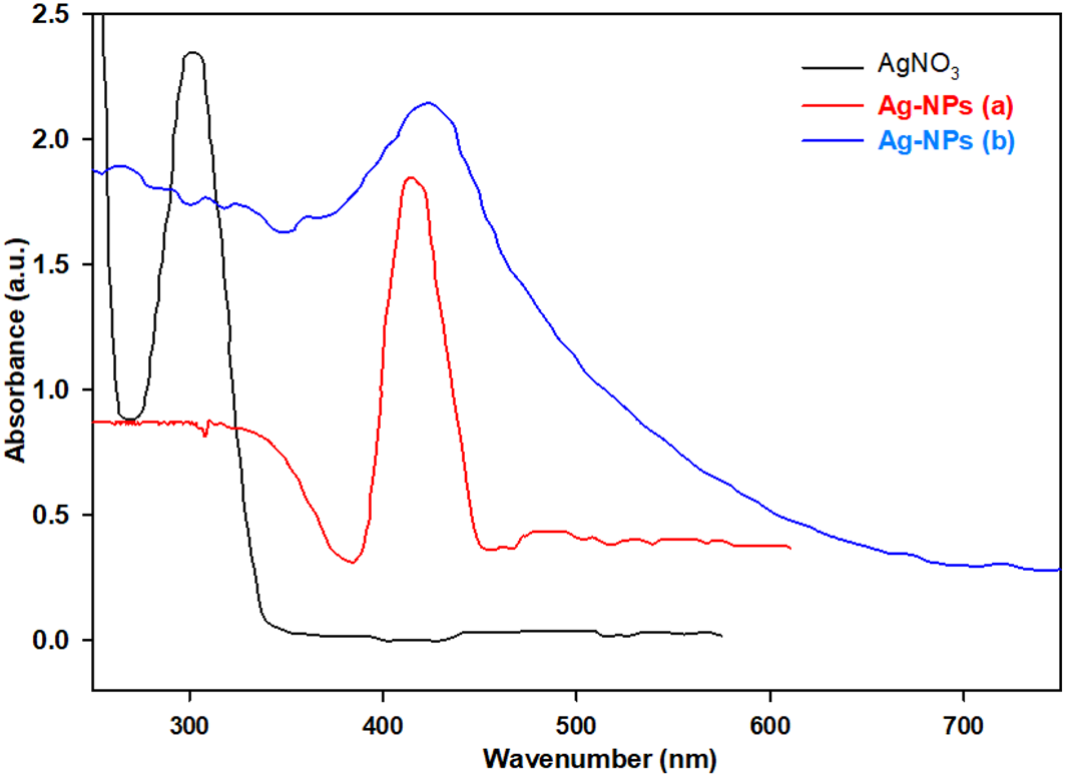
UV–vis spectra of AgNO_3_ and synthesized Ag-NPs in the presence of (a) *Thyme leaves extract* and (b) NaBH_4_ (0.1 M).

**Figure 8:**
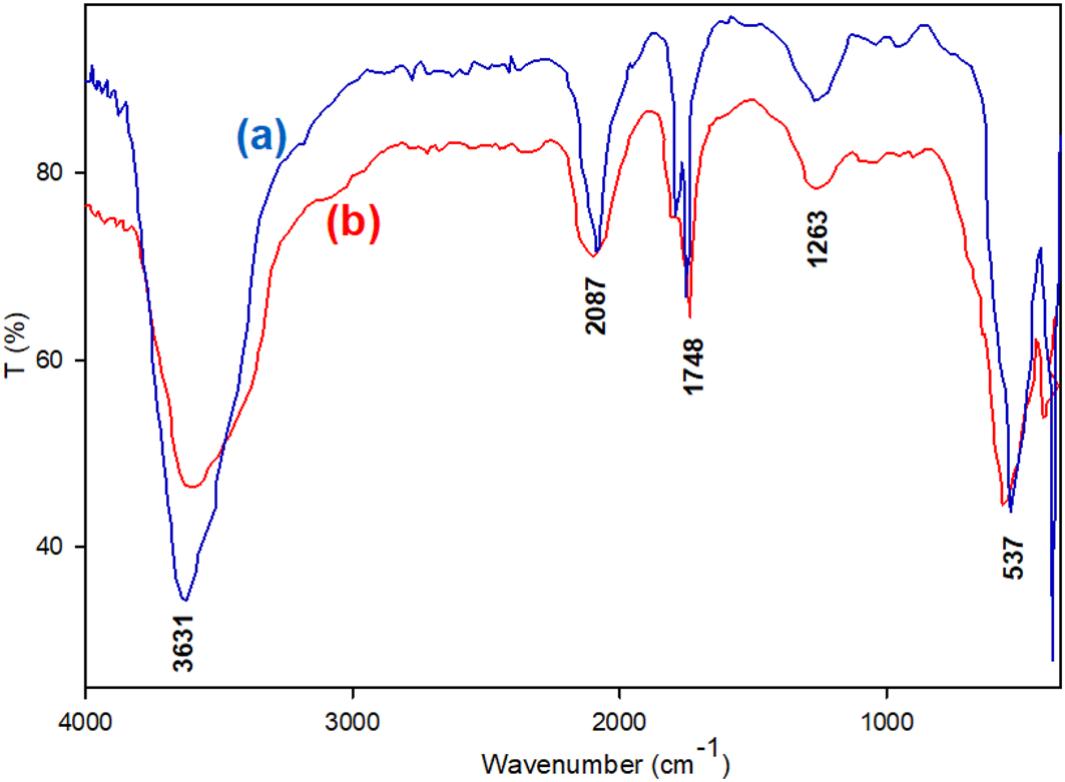
FT-IR of synthesized Ag-NPs in the presence of *Thyme leaves extract* (a) and NaBH_4_ (0.1 M) (b).

As evidenced by the results obtained in Table 4 (Entry 6), the AgNO_3_ compound demonstrates efficient activity for the degradation of OPDs. Based on the available literature^51,52^, the combination of silver nitrate and sodium borohydride has been found to facilitate the formation of reduced Ag-NPs. The experimental results suggest that initially, Ag-NPs are produced, and subsequently, they play a catalytic role in the degradation of the desired OPDs. Because of the challenge lies in their limited recyclability, the lack of a suitable substrate hinders the optimal performance of this conditions (Table 4, entry 6). In this section, the preparation of Ag-NPs was investigated using two separate solutions (*Thyme plant leaves* and NaBH_4_ solution) containing silver reducing agents. Silver nitrate solution (20 mL, 0.02 M) was combined with either 10 mL of *Thyme* plant leaves or 50 mL of NaBH_4_ solution (0.1 M). The resulting mixture was magnetically stirred (1200 rpm) in a 250 mL round-bottom flask at 80 °C for 8 h under dark conditions. Subsequently, the variations in absorbance of the solution were tracked through the utilization of UV–vis spectroscopy and the produced precipitate was separated by centrifugation (8000 rpm) and dried overnight at 100 °C. The synthesized Ag-NPs were then characterized using FT-IR spectroscopy. The analysis of the synthesized Ag-NPs revealed peaks that aligned with the findings reported in literature^27,48,53^ (Figs. 7, 8).

By the findings presented in Table 4, it is indicated that the OPD degradation reaction cannot be adequately catalyzed by either silver nitrate alone (Table 4, entry 7) or in combination with zeolite (Table 4, entry 8). The crucial role of sodium borohydride is underscored in facilitating the desired reaction.

In our pursuit of environmentally friendly processes and the degradation of environmental pollutants, we have neglected to explore its potential as a catalyst in the OPD degradation reaction. As a result, all subsequent reactions to determine optimal conditions were carried out with the concurrent presence of NaBH_4_ and Ag@Z-5 (Table 5).

**Table 5:** Optimization of catalytic degradation conditions of OPDs (50 ml, 50 ppm) in the simultaneous presence of Ag@Z-5 and NaBH_4_.

Entry	Ag@Z (mg)	NaBH_4_ (mL)	MB	4-NP	MO	4-NA
			Time/Deg.	Time/Deg.	Time/Deg.	Time/Deg.
1	5	40	3/98	4/99	6/96	7/94
2	5	30	3/99	4/98	8/95	8/94
3	5	20	3/98	4/98	8/94	15/87
4	5	10	3/98	5/97	15/81	15/81
5	4	10	4/98	**5/97**	–	–
6	3	10	**5/98**	7/92	–	–
7	2	10	8/93	15/89	–	–
8	1	10	15/94	20/87	–	–
9	4	20	–	–	**8/94**	–
10	3	20	–	–	10/85	–
11	2	20	–	–	15/80	–
12	1	20	–	–	20/76	–
13	4	30	–	–	–	**8/93**
14	3	30	–	–	–	15/86
15	2	30	–	–	–	20/82
16	1	30	–	–	–	30/75

Optimum conditions are bolded.

The experimental results confirm our initial expectations by demonstrating the positive influence of increasing the dosage of Ag@Z-5 and NaBH_4_ on the reaction rate and efficiency. The augmentation of Ag@Z-5 and NaBH_4_ dosages enhances the availability of reaction sites on the catalyst's surface, leading to an improved overall efficiency of OPD degradation and an apparent increase in the reaction rate. However, it is important to note that the catalytic degradation efficiency of OPDs reaches a saturation point once the catalyst (or NaBH_4_) dosage exceeds a certain threshold. To identify the optimal degradation conditions, we conducted separate experiments for each OPD, varying the dosages of NaBH_4_ (0.1 M, 10–40 mL) and Ag@Z-5 (1–5 mg).

The results obtained, presented in Table 5, provide valuable insights into the degradation of the target dye. It is evident from the results that there is a clear correlation between the degradation of the dye and the reduction in intensity at its maximum absorption wavelength. Notably, the maximum absorption wavelengths for MB, MO, 4-NP, and 4-NA dyes are 663, 465, 400, and 380 nm, respectively. It is worth mentioning that the 4-NP dye initially exhibited a maximum absorption at 315 nm. However, in the presence of NaBH_4_, the maximum absorption peak of 4-NP shifted to 400 nm. This shift in absorption wavelength can be attributed to the conversion of 4-NP to 4-nitrophenolate ion in the presence of sodium borohydride^43,54^. Therefore, in the evaluation and assessment of the catalytic degradation reaction of 4-NP, changes in absorption intensity at the 400 nm wavelength were utilized as the primary parameter. Table 5 provides a summary of the specific dosages of Ag@Z-5 and NaBH_4_ that were employed to determine the optimal reaction conditions for the degradation of MB, MO, 4-NP, and 4-NA dyes.

**Figure 9:**
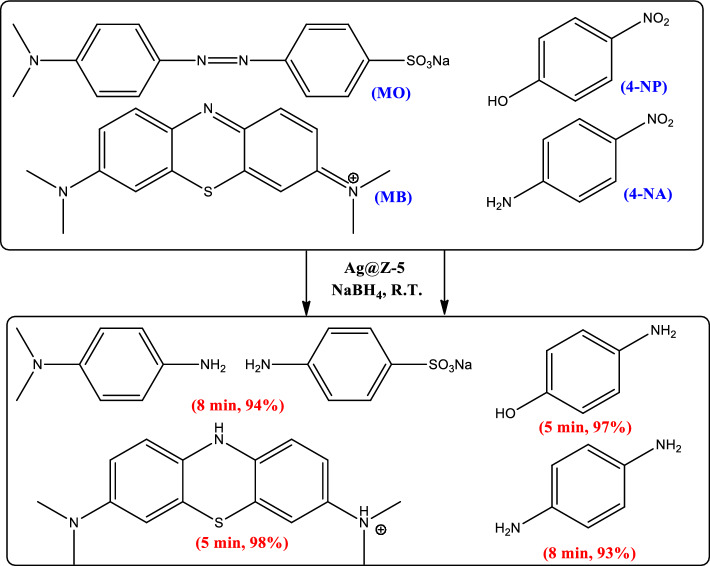
Schematic degradation of OPDs.

The results demonstrate that the efficient degradation of the organic dye pollutants under investigation can be achieved within a short timeframe (5–8 min) under optimized conditions, with an impressive efficiency exceeding 93%. These findings suggest that the Ag@Z-5 composite can serve as a highly effective catalyst for the efficient degradation of organic dye pollutants present in real wastewater samples. Schematic degradation for the investigated organic dyes is depicted in Fig. 9.

Following that, the reaction kinetics of each OPD were assessed under the determined optimal degradation conditions. The focus of this study was to investigate the kinetics of dye degradation reactions by analyzing the decrease in absorption intensity at their respective maximum wavelengths. This approach was chosen due to the direct correlation between the change in absorption intensity and the percentage of degradation reaction progress. The following equation was employed in this study to calculate the reaction efficiency of dye degradation in the presence of Ag@Z-5:

$$Degradation (\%)=\left(\frac{\left[{A}_{o}\right]- \left[{A}_{o}\right]}{\left[{A}_{o}\right]}\right)\times 100.$$

In order to examine the kinetics of OPD degradation, a concentration–time graph was constructed for each OPD. Since the concentration of NaBH_4_ (0.1 M) was significantly higher than that of the OPDs, the reduction process was treated as a pseudo-first-order reaction. The variables [C_o_] and [A_o_] represented the initial concentration and absorbance intensity, respectively, while [C_t_] and [A_t_] denoted the concentration and absorbance intensity at different reaction times. By utilizing the equation and the relationship between absorbance and concentration, a ln ([C_t_]/[C_o_]) vs. time graph was plotted. The obtained data points were then fitted to a linear trend line to determine the reaction rate constant (*k*_*app*_, min^–1^).

$$ ln\left( {A_{t} /A_{o} } \right) \, = \, ln \, \left( {\left[ {C_{t} } \right]/\left[ {C_{o} } \right]} \right) \, = - kt . $$

**Figure 10:**
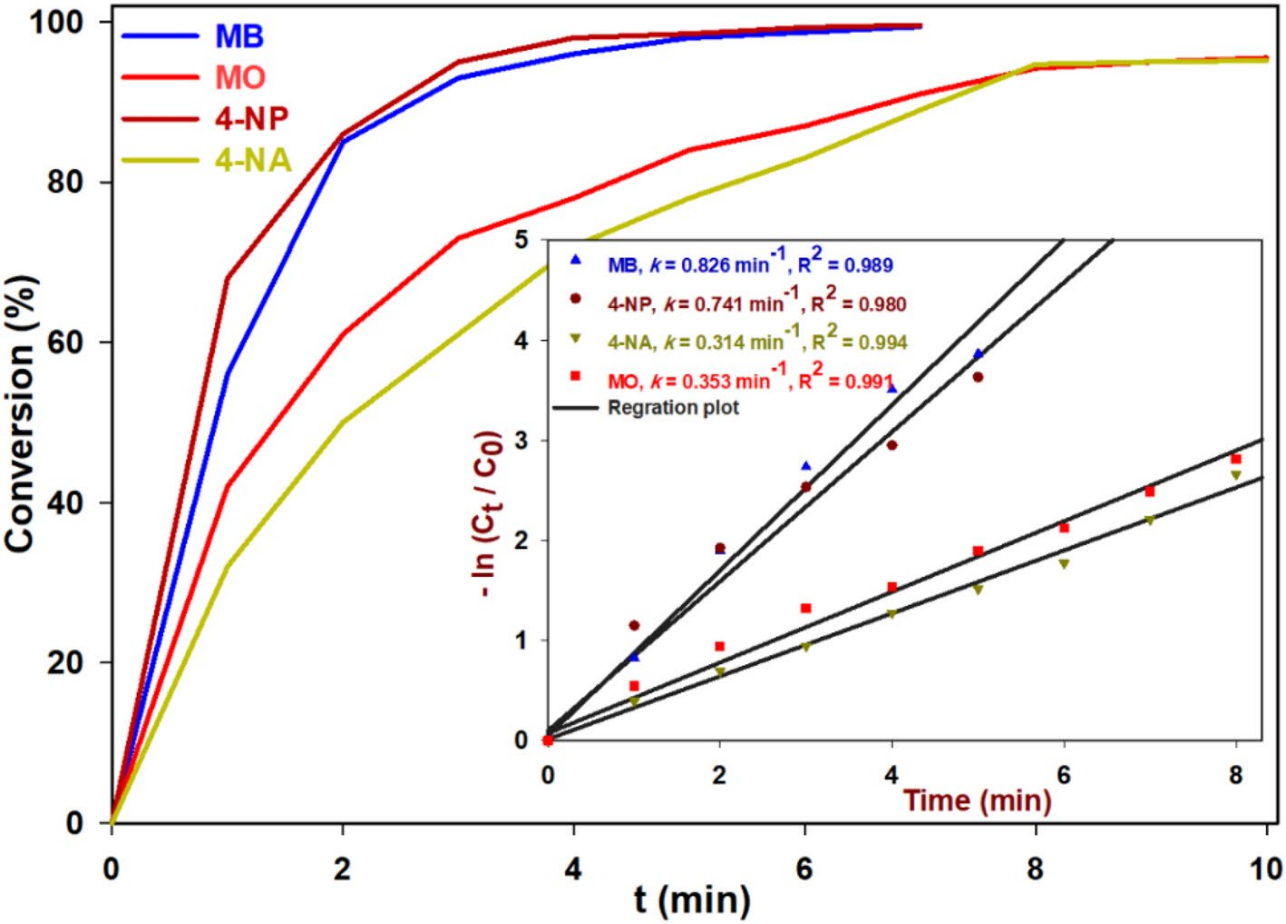
Kinetic study of degradation reaction of OPDs (MB, 4-NP, 4-NA and MO).

The kinetics of organic dye degradation were investigated under optimized conditions, focusing on the rate constants of the degradation reactions. The results showed different rate constants for the degradation of each dye: MB, 4-NP, 4-NA and MO have rate constants of 0.826, 0.741, 0.314, and 0.353 min^–1^, respectively (Fig. 10).

The aim of this study was to employ Ag@Z-5 as a novel and efficient catalyst for the catalytic degradation of OPDs in the presence of NaBH_4_ solution. The obtained results were then compared to findings from similar studies, highlighting the exceptional effectiveness of the catalyst. It demonstrated a significantly higher level of degradation progress, an increased reaction rate, and a reduced catalyst dosage-to-dye ratio. These findings emphasize the superior advantages offered by this catalyst compared to alternative catalysts used in previous research (Table 6).

**Table 6:** Comparison of catalytic reduction of MB and 4-NP in the presence of various catalysts.

OPD	Catalyst (mg)	Dye (mL, ppm)	NaBH_4_ (mL, M)	Time (min), k (min^–1^)	Ref
MB	MPD-Cu (0.4)^a^	0.1, 200 ppm	(0.66, 3 M)	5, 1.44	^55^
	Cu(NPs)/β-CCP (10)^b^	2, 166 ppm	(0.50, 0.04 M)	4, 0.57	^56^
	AgMoOS (10)	100, 20 ppm	(2.0, 0.2 M)	6, 0.541	^57^
	NC-AgNPs (50)	2, 20 ppm	(0.95, 0.005 M)	150, 0.16	^58^
	Ag@Z-5 (3)	50, 50 ppm	(10, 0.1 M)	5, 0.826	This work
4-NP	Co/PCNS (0.1)	2, 20 ppm	(11, 0.125 M)	7, 0.31	^59^
	AgMoOS (10)	100, 20 ppm	(2, 0.2 M)	18, 0.136	^57^
	Cu-NP/C (4.0)	1.5, 27.8 ppm	(1.5, 0.02 M)	6, 0.3	^60^
	Cu/MC (0.5)	6, 42 ppm	(2, 0.5 M)	5, 0.96	^61^
	Ag@Z-5 (4)	50, 50 ppm	(10, 0.1 M)	5, 0.741	This work

^a^Magnetic polydopamine-Cu nanoflowers.

^b^Cu(NPs)/β-Chitin/dicalcium phosphate.

### Evaluating the Ag@Z-5 catalyst’s recyclability and reusability

**Figure 11:**
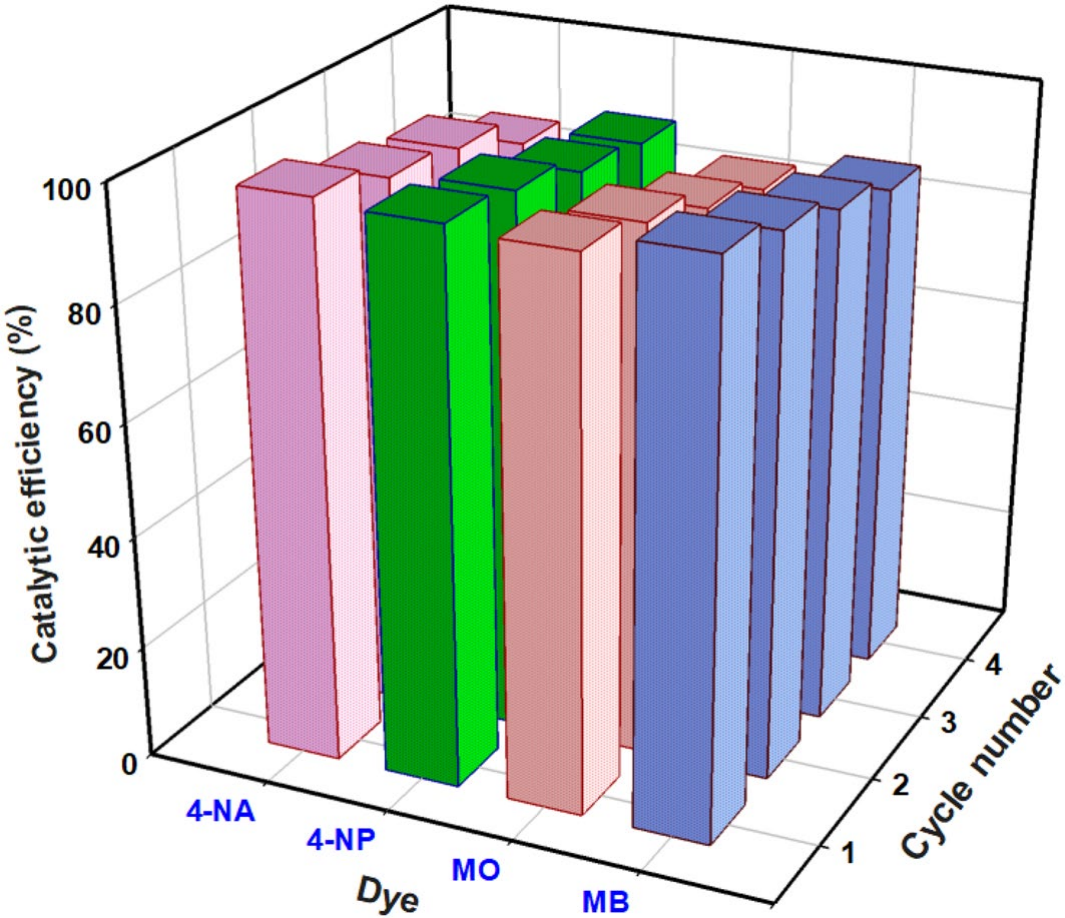
Catalytic efficiency of recycled Ag@Z-5.

From an environmental perspective, catalysts that have better chemical stability, recyclability, and reusability are particularly important and also hold economic significance. These properties are crucial for the practical utilization of catalysts. Although the main objective of researchers in this study is the removal of pollutants and the presentation of efficient conditions for water treatment, it should be noted that the importance of catalyst recyclability in environmental preservation, waste reduction, and energy consumption cannot be underestimated. Therefore, in this study, we investigated the recoverability and reusability of the used catalyst. After the completion of the dye degradation reaction, the used catalysts were collected using filtration, centrifugation, washed, and dried overnight. The catalyst's efficiency in this study is directly impacted by the concentration of silver nanoparticles in its composite. Our findings indicate that the recycled catalyst significantly decreases in efficiency by approximately 10–15% after four recovery steps (Fig. 11). These results emphasize the strong interactions between the silver nanoparticles and the zeolite matrix, which effectively prevent silver leaching during the recycling and cleaning processes. Consequently, this improves the stability and longevity of the catalyst, emphasizing its substantial implications in the respective domain.

Additionally, different conditions were explored and compared for the immobilization of silver nanoparticles using solid supports, specifically amorphous zeolite (am-Z) and powder Alumino-Silicate glass (AS-glass). These supports were considered as alternatives to the synthesized zeolite. The silver nanoparticles were prepared and subsequently immobilized onto the solid supports under similar conditions (Ag@am-Z and Ag@AS-glass). The performance of Ag@am-Z and Ag@AS-glass in the degradation reaction of 4-NP was then investigated. It was found that the efficiency of Ag@am-Z and Ag@AS-glass in the degradation of 4-NP was comparable to that of the Ag@Z-5 catalyst in terms of yield. However, a significant weakness and irreversible decrease in the efficiency of the recycled Ag@am-Z and recycled Ag@AS-glass were observed. After recycling, the efficiency decreased by 40–61%. This indicates a decline in the effectiveness of Ag@am-Z and Ag@AS-glass after multiple recycling steps. The superior stability and immobilization of silver nanoparticles on the surface of the synthesized zeolite were suggested by the results. These findings hold vital implications for the development of more efficient catalysts for various applications (Supplementary Information).

## Conclusion

In this study, *Thyme leaves extract* was utilized as an eco-friendly reducing agent for the synthesis of Ag-NPs. The reduction of AgNO_3_ was facilitated by the phytochemical compounds present in the *Thyme leaves extract*, eliminating the need for harsh chemicals. The NPs were immobilized on synthesized zeolite, resulting in the dispersion of stable Ag-NPs on the external surface of the zeolite. The structure and morphology of the as synthesized Ag@Z-5 was characterized using XRD, FESEM, EDS and TEM; revealing that the zeolite’s structure and crystallinity were not significantly altered by the coating of NPs. Additionally, the potential of the synthesized Ag@Z-5 as catalysts for the reduction of OPDs (MB, 4-NP, 4-NA and MO) using NaBH_4_ solution (0.1 M) was evaluated. Increasing the catalyst concentration led to an enhancement in the reaction rate by providing more active sites. The synthesized Ag@Z-5 exhibited efficient degradation of OPDs within a short time period (5–8 min), with an efficiency exceeding 93% at optimized conditions. The degradation kinetics of each dye were assessed by monitoring changes in maximum absorption intensity, and the rate constant for the degradation reaction (*k*_*app*_, min^–1^) was calculated. The reusability of the catalyst was also examined. The catalyst could be reused four times without significant loss in catalytic activity, likely due to thorough washing and removal of Ag-NPs. Future studies will focus on investigating the photocatalytic properties of Ag@Z-5 for the degradation of wastewater pollutants.

### Supplementary Information

Supplementary Information.

## Data Availability

The datasets used and/or analyzed during the current study are available from the corresponding author at reasonable request.
