# Supplementary material for: In situ and bio-green synthesis of silver nanoparticles immobilized on zeolite as a recyclable catalyst for the degradation of OPDs
Source: Sci Rep. 2024 Jan 11;14:1143. doi: 10.1038/s41598-024-51271-9 (PMC10784553; doi:10.1038/s41598-024-51271-9)
Supplement: Supplementary file 1 — Supplementary Information. [file 41598_2024_51271_MOESM1_ESM.docx]

**In situ and bio-green synthesis of silver nanoparticles immobilized on zeolite as a recyclable catalyst for the degradation of OPDs**

Fujiang Zhou^1^, Danfeng He^1^*, Guojian Ren^2^ and Hossein Yarahmadi^3^*

*^1^College of Science, Qiongtai Normal University; Haikou 571100, Hainan, China*

*^2^Key Laboratory of Advanced Materials of Tropical Island Resources, Ministry of Education, School of Chemistry and Chemical Engineering, Hainan University, Haikou 570228, Hainan, China*

*^3^Department of Chemical Engineering, Sirjan University of Technology, Sirjan, Iran*

*^*^Corresponding authors: Email: Hyarahmadi61@gmail.com (Hossein Yarahmadi);Hedanfeng@mail.qtnu.edu.cn (Danfeng He)*

**Supporting information data**

1. *Efficiency of Thyme leaves extract to reduction of silver ions*

The effectiveness of the *Thyme leaves extract* solution in reduction reaction of silver ions was investigated by AgNO_3_ solution (0.02 M). A 250 ml round bottom flask fitted with a water condenser was utilized. Specifically, 10-20 ml of the extracted solution was added to AgNO_3_ solution (20-150 ml, 0.02 M) and the resulting mixture was stirred magnetically (1200 rpm) in darkness conditions at 100 °C for 8 hours. The concentration of silver ions in the solution was analyzed using the maximum wavelength of silver nitrate through UV-vis analysis (Figure S1). As the concentration of silver ions decreased, the peak intensity at 300 nm was decreased, while the peak absorption intensity associated with Ag-NPs was simultaneously increased. Under the specified conditions, it was found that 10 ml of the *Thyme leaves extracted* solution had the capacity to reduce 0.0012 moles of silver ions.

| **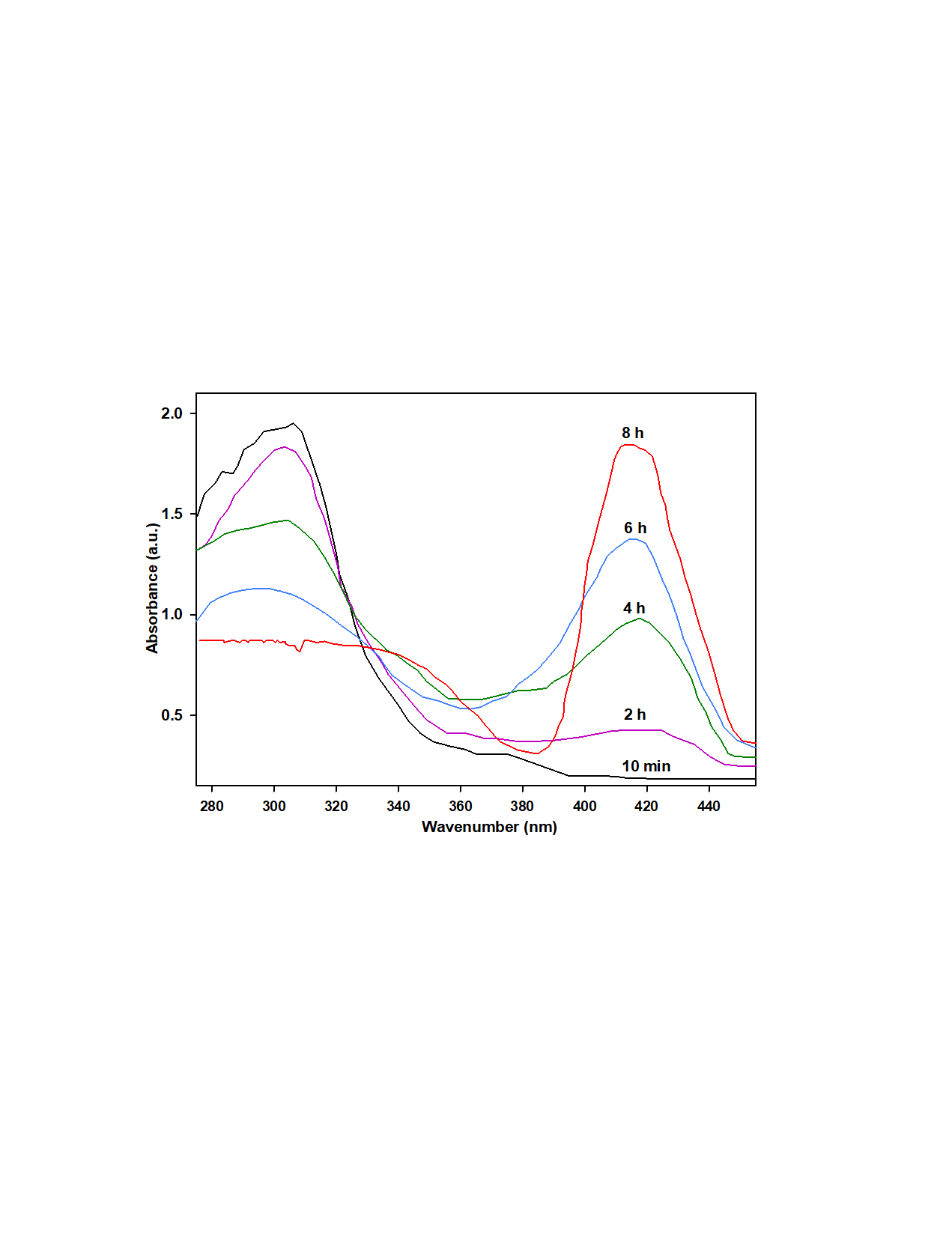** |
| --- |
| **Figure S1.** UV–Vis spectra of synthesized AgNPs using AgNO_3_ in the presence of *Thyme* leaves extract. |

1. *The catalytic degradation of OPDs (MB, MO, 4-NP and 4-NA) in the presence of Ag@Z-5 and NaBH_4_ at optimized conditions*

In order to examine the kinetics of OPD degradation at optimized conditions, a concentration-time graph was constructed for each OPD (MB, MO, 4-NP, and 4-NA) as followed:

| **Degradation of MB**   \| Time (mine) \| Degradation (%) \| \| --- \| --- \| \| 1 \| 56 \| \| 2 \| 85 \| \| 3 \| 93 \| \| 4 \| 96 \| \| 5 \| 98 \| \| 6 \| 98 \| \| 7 \| 99 \| | 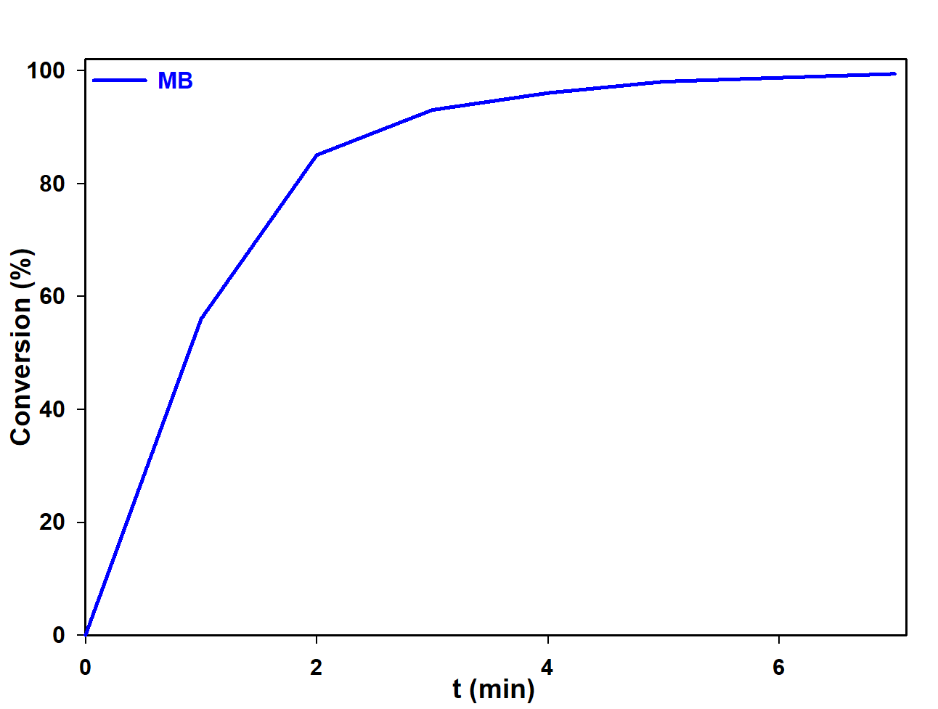 |
| --- | --- | --- | --- | --- | --- | --- | --- | --- | --- | --- | --- | --- | --- | --- | --- | --- | --- |
| **Figure S2**. Degradation of MB at optimized conditions (MB: 50 ml, 50 ppm; Catalyst (Ag@Z-5): 3 mg; NaBH_4_: 10 ml, 0.1 M) | |

| **Degradation of MO**   \| Time (mine) \| Degradation (%) \| \| --- \| --- \| \| 1 \| 42 \| \| 2 \| 61 \| \| 3 \| 73 \| \| 4 \| 78 \| \| 5 \| 84 \| \| 6 \| 87 \| \| 7 \| 91 \| \| 8 \| 94 \| \| 9 \| 95 \| \| 10 \| 95 \| | 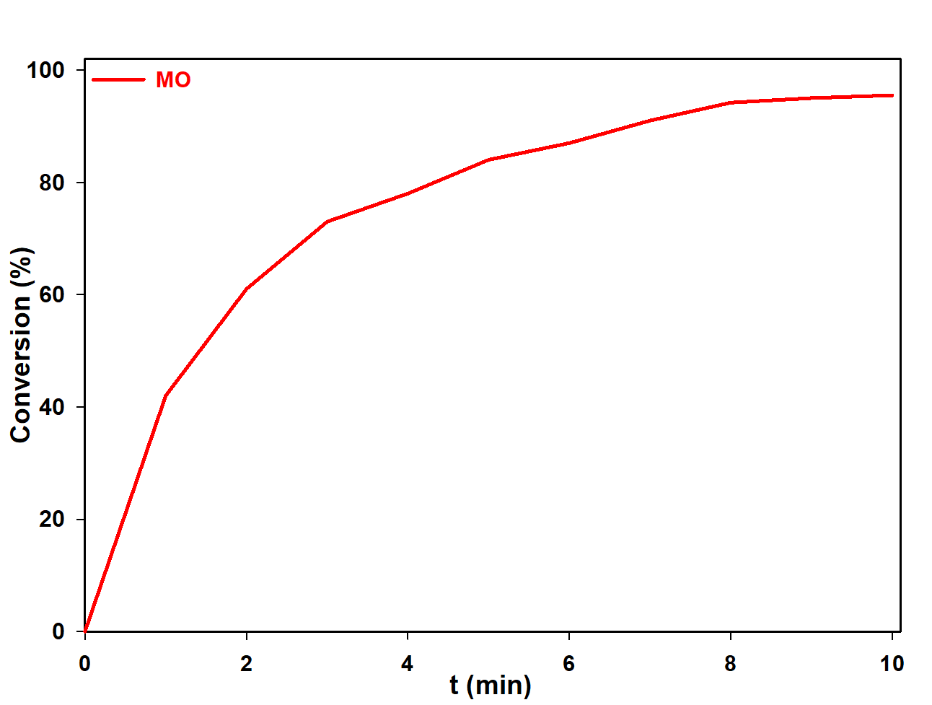 |
| --- | --- | --- | --- | --- | --- | --- | --- | --- | --- | --- | --- | --- | --- | --- | --- | --- | --- | --- | --- | --- | --- | --- | --- |
| **Figure S3**. Degradation of MO at optimized conditions (MO: 50 ml, 50 ppm; Catalyst (Ag@Z-5): 4 mg; NaBH_4_: 20 ml, 0.1 M) | |

| **Degradation of 4-NP**   \| Time (mine) \| Degradation (%) \| \| --- \| --- \| \| 1 \| 68 \| \| 2 \| 86 \| \| 3 \| 95 \| \| 4 \| 98 \| \| 5 \| 98 \| \| 6 \| 99 \| \| 7 \| 99 \| | 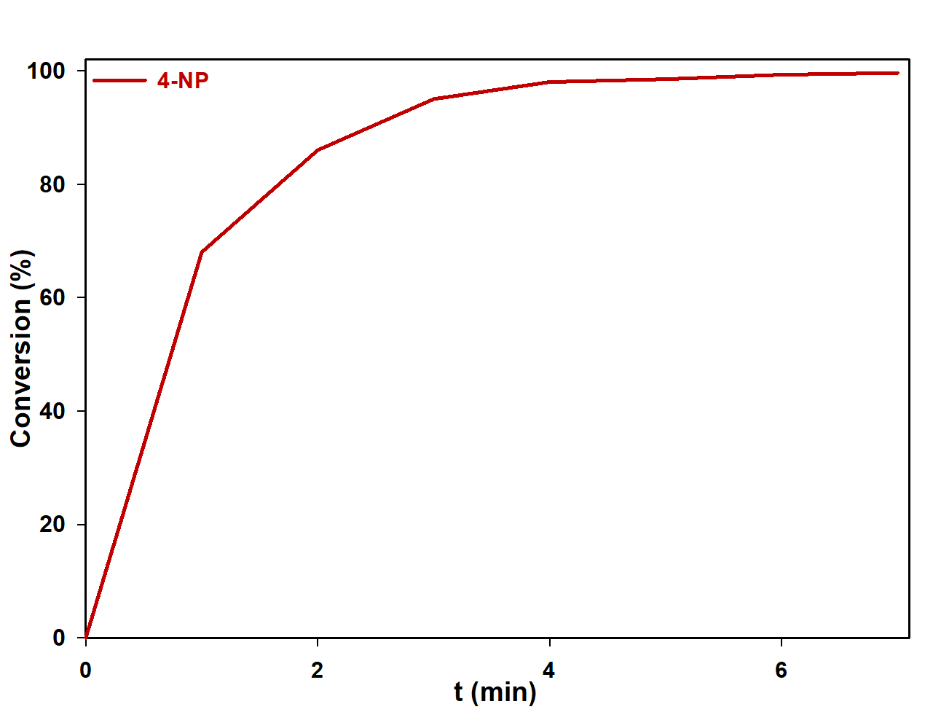 |
| --- | --- | --- | --- | --- | --- | --- | --- | --- | --- | --- | --- | --- | --- | --- | --- | --- | --- |
| **Figure S4**. Degradation of 4-NP at optimized conditions (4-NP: 50 ml, 50 ppm; Catalyst (Ag@Z-5): 4 mg; NaBH_4_: 10 ml, 0.1 M) | |

| **Degradation of 4-NA**   \| Time (mine) \| Degradation (%) \| \| --- \| --- \| \| 1 \| 32 \| \| 2 \| 50 \| \| 3 \| 61 \| \| 4 \| 72 \| \| 5 \| 78 \| \| 6 \| 83 \| \| 7 \| 89 \| \| 8 \| 94 \| \| 9 \| 95 \| \| 10 \| 95 \| | 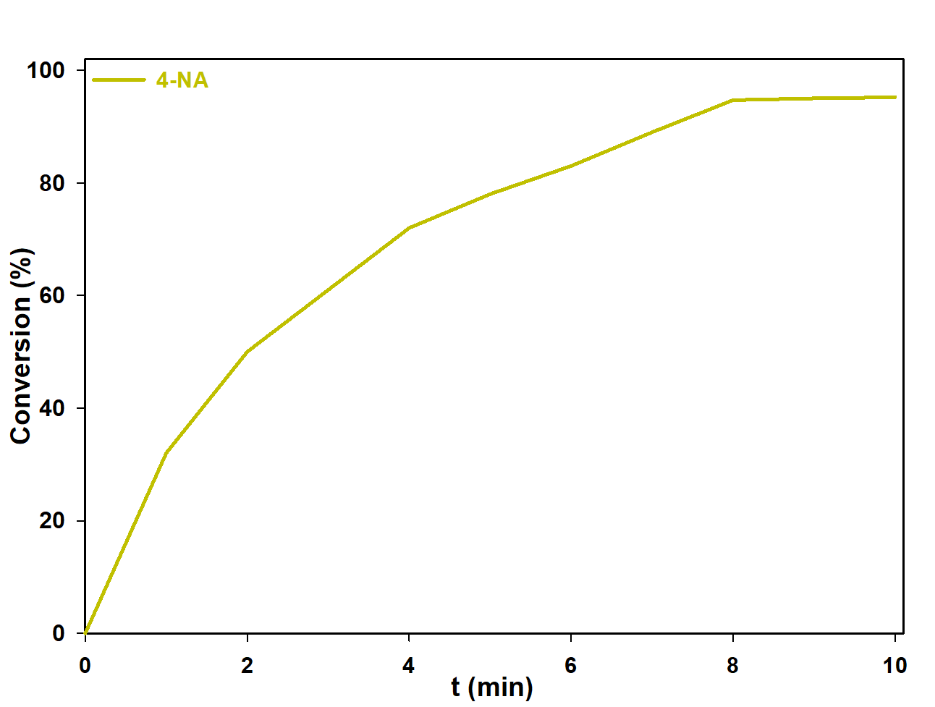 |
| --- | --- | --- | --- | --- | --- | --- | --- | --- | --- | --- | --- | --- | --- | --- | --- | --- | --- | --- | --- | --- | --- | --- | --- |
| **Figure S5**. Degradation of 4-NA at optimized conditions (4-NA: 50 ml, 50 ppm; Catalyst (Ag@Z-5): 4 mg; NaBH_4_: 30 ml, 0.1 M) | |
